# Nephrotoxicity and Modern Volatile Anesthetics: A Narrative Review

**DOI:** 10.3390/toxics13060514

**Published:** 2025-06-19

**Authors:** Benedicte Hauquiert, Aurelien Gonze, Thibault Gennart, Emily Perriens, Sydney Blackman, Nathan De Lissnyder, Arnaud Robert, Julien Moury, Gauthier Nendumba, Ilann Oueslati, Priscilla Gillis, Ovidiu Vornicu, Anne-Sophie Dincq, Pierre Bulpa, Isabelle Michaux, Patrick M. Honore

**Affiliations:** 1 ICU, CHU UCL Godinne Namur, UCL Louvain Medical School, 5530 Yvoir, Belgium; benedicte.hauquiert@gmail.com (B.H.); aurelien.gonze@chuuclnamur.uclouvain.be (A.G.); thibault.gennart@chuuclnamur.uclouvain.be (T.G.); arnaud.robert89@gmail.com (A.R.); julien.moury@chuuclnamur.uclouvain.be (J.M.); gauthier.nendumba@chuuclnamur.uclouvain.be (G.N.); priscilla.gillis@gmail.com (P.G.); pierre.bulpa@chuuclnamur.uclouvain.be (P.B.); isabelle.michaux@chuuclnamur.uclouvain.be (I.M.); 2 Faculty of Medicine, ULB University, 1070 Brussels, Belgium; emily.perriens@ulb.be (E.P.); ilann.oueslati@ulb.be (I.O.); 3 Department of Gynecology, Centre Hospitalier Interregional Edith Cavell (Chirec Hospital), 1180 Brussels, Belgium; blackman.sydney@gmail.com; 4 Department of Surgery, Vrije Universiteit Brussel, UZ Brussel, 1090 Brussels, Belgium; 5 ICU and Anesthesiology Departments, CHU UCL Godinne Namur, UCL Louvain Medical School, 5530 Yvoir, Belgium; ovidiu.vornicu@chuuclnamur.uclouvain.be (O.V.); anne-sophie.dincq@chuuclnamur.uclouvain.be (A.-S.D.)

**Keywords:** volatile anesthetics, sevoflurane, inorganic fluoride, nephrotoxicity, nephrogenic diabetes insipidus

## Abstract

Volatile anesthetics, while increasingly utilized in intensive care medicine, are associated with significant renal adverse effects. A critical safety concern—particularly with sevoflurane—involves its potential impact on renal function. Pathophysiologically, inorganic fluoride levels exceeding 50 µmol/L are recognized as a threshold for nephrogenic diabetes insipidus, a condition generally considered reversible. Additionally, the sevoflurane degradation product “compound A” has been implicated in direct renal tubular and glomerular toxicity. Specifically, exposure has been correlated with glomerular damage, evidenced by albuminuria, as well as injury to both proximal and distal tubules, indicated by elevated levels of α-glutathione-S-transferase. Postprandial glycosuria may also be observed. Unlike nephrogenic diabetes insipidus, the structural damage induced by compound A may result in irreversible renal impairment.

## 1. Introduction

Since Pringle et al. first described oliguria during ether anesthesia in 1905, numerous studies have investigated the effects of anesthetic agents on renal function [1]. Volatile anesthetics are known to induce significant renal adverse effects and are widely used in both operating rooms (ORs) and intensive care units (ICUs). This review aims to elucidate their nephrotoxic potential, underlying mechanisms, reversibility of renal injury, and possible therapeutic interventions—including the existence of a therapeutic window [2].

A distinction must be made between older and contemporary volatile agents. Most modern volatile anesthetics are halogenated methyl ethyl esters, with the exception of halothane, a fluorinated alkane. Methyl ethyl ester anesthetics exhibit greater potency and improved anesthetic profiles compared to diethyl ethers. Historically, methoxyflurane was associated with significant nephrotoxicity due to excessive fluoride ion production, ultimately leading to its withdrawal from clinical use. Although newer agents were initially considered renally safe, contemporary anesthetics—particularly sevoflurane—can still produce nephrotoxic fluoride concentrations. Recent reports have documented renal adverse effects associated with sevoflurane and other modern volatile agents [3]. Currently, commonly used volatile anesthetics include fluorinated liquids such as isoflurane, desflurane, and sevoflurane [4].

**Figure 1:**
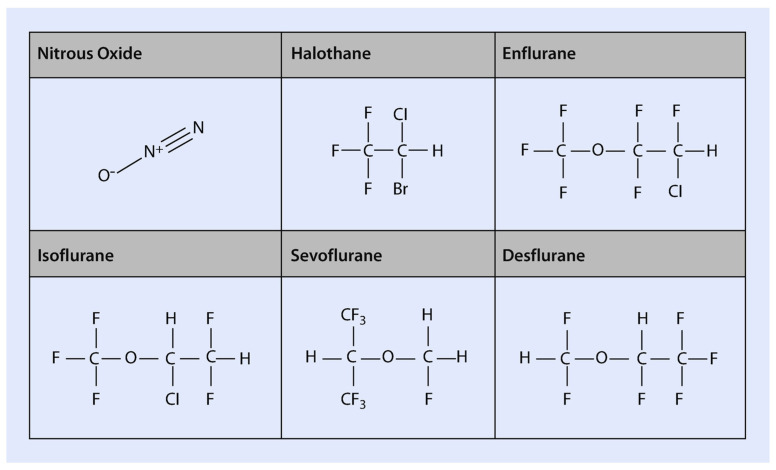
Chemical structure of inhaled anesthetics. Halothane is an alkane, a halogen-substituted ethane derivative. Isoflurane and enflurane are isomers that are methyl-ethyl ethers. Desflurane differs from isoflurane in the substitution of a fluorine for a chlorine atom, and sevoflurane is a methyl isopropyl ether.

**Figure 2:**
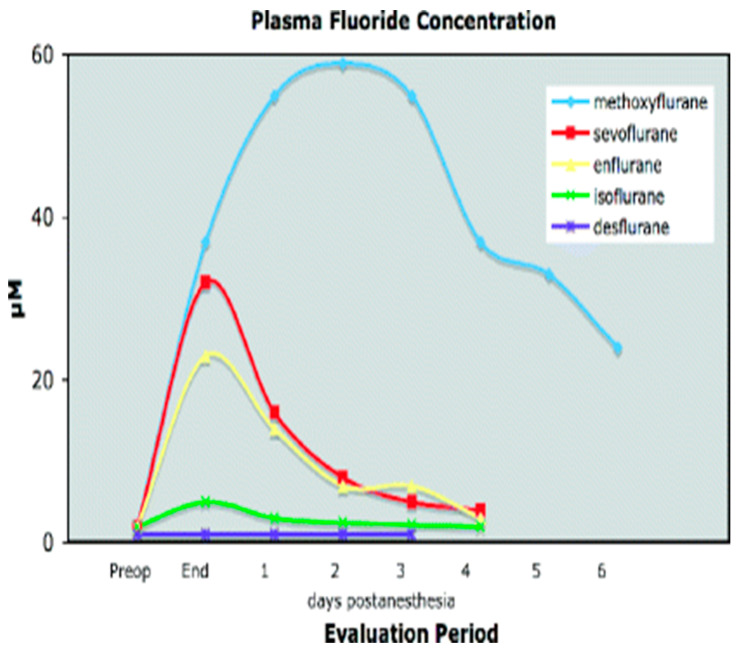
Plasma inorganic fluoride concentrations (mean ± SEM) before and after 2–4 h of methoxyflurane, enflurane, sevoflurane, isoflurane, and desflurane anesthesia.

Fluoride inhibits several enzyme systems, impairing tissue respiration and anaerobic glycolysis [5]. In the kidneys, fluoride disrupts sodium transport in the proximal convoluted tubule, inhibits adenylate cyclase, and diminishes the action of antidiuretic hormone. Experimental evidence in rats indicates that fluoride also inhibits the chloride pump in the thick ascending limb of Henle’s loop. Furthermore, the duration of fluoride exposure—dependent on its production and elimination—plays a critical role in the development of nephrotoxicity [6] (Figure 1 and Figure 2).

### 1.1. Metabolism of Volatile Anesthetics

Modern inhalation anesthetics are fluorinated to reduce flammability. Initially believed to be biochemically inert, it is now recognized that these agents undergo in vivo metabolism [7], with their metabolites responsible for both acute and chronic toxicities [8,9]. Research over the past 25 years has led to changes in clinical practice, including the discontinuation of methoxyflurane due to nephrotoxicity and restricted use of halothane due to hepatotoxicity. These findings have also driven the development of newer agents, such as isoflurane and desflurane, designed to minimize toxic potential. Despite these advances, further improvements are needed as our understanding of toxicological mechanisms evolves.

The cytochrome P-450 enzyme system mediates the initial metabolism of inhaled anesthetics, primarily via oxidation. Some agents, such as halothane, may also undergo reductive metabolism under certain conditions. Additionally, certain anesthetics (e.g., sevoflurane) undergo phase II conjugation reactions prior to excretion. The cytochrome P-450 system comprises multiple inducible isoenzymes, with induction influenced by factors such as exposure to ethanol, phenobarbital, cimetidine, phenytoin, isoniazid, and even volatile anesthetics themselves [10,11]. Induction involves stimulation of transcriptional and translational processes, increasing cytochrome P-450 production [12]. Isoenzyme expression is also modulated by sex, obesity, fasting, and diabetes. For example, streptozotocin-induced diabetes in rats increases P-450 IIE1 expression several-fold, enhancing enflurane and isoflurane metabolism [13].

**Figure 3:**
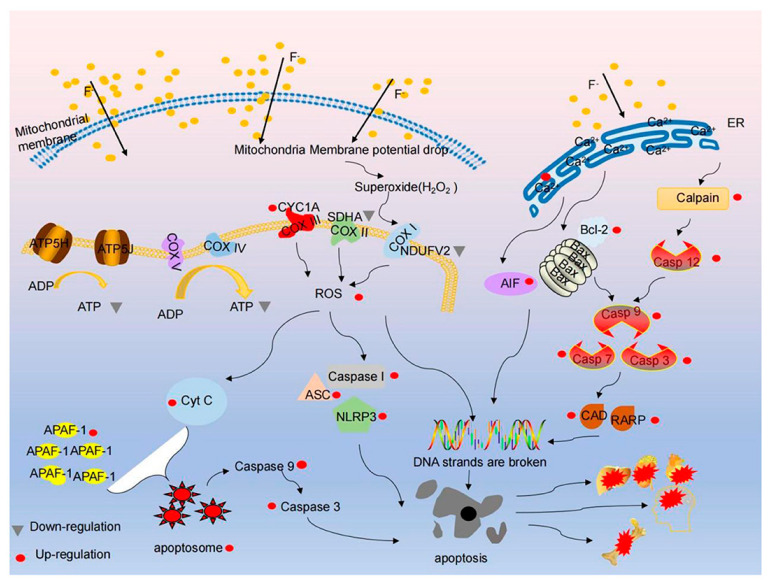
Effects of fluoride on cytoxicity involved in mitochondrial dysfunction.

Although halogenated anesthetics share structural similarities, their metabolic rates and pathways vary significantly. Minor structural alterations can lead to major changes in metabolism. Lipid solubility, which determines drug access to metabolizing enzymes and duration of exposure, also plays a key role in metabolic rate and biotransformation [14] (Figure 3).

### 1.2. Nephrotoxic Effects

In animal models, sevoflurane degradation, which produces compound A, has been associated with proximal tubular necrosis. However, definitive cases of such injury directly attributable to sevoflurane have not been established in humans [15]. Another proposed mechanism involves subclinical nephrotoxicity mediated by lipid and protein oxidation [16].

Nephrogenic diabetes insipidus (NDI) is a recognized complication of prolonged volatile anesthetic exposure in ICUs [17]. A recent study observed NDI in 7 of 25 patients receiving sevoflurane for >72 h [18]. While polyuria typically resolved within 48 h, complete recovery was not universal [7,18]. The impaired urinary concentrating ability was attributed to two mechanisms: sevoflurane-induced disruption of aquaporin expression and fluoride accumulation. A plasma fluoride threshold of 50 µmol/L is considered nephrotoxic, based on prior observations with methoxyflurane [8,19]. No long-term renal sequelae were reported in that cohort [20].

Fluoride is cleared via urinary excretion (50%) and uptake into calcified tissues (50%) [21,22,23,24]. Renal fluoride excretion involves glomerular filtration with variable tubular reabsorption, influenced by tubular fluid flow rate [25] and urinary pH [26,27]. Alkaline urine reduces plasma fluoride levels by 50% compared to acidic urine [28]. Bone uptake also modulates fluoride concentrations; metabolic acidosis increases bone resorption, while alkalosis enhances osseous accretion [29].

Sevoflurane undergoes extensive defluorination but, due to low lipid solubility, is rapidly eliminated post-anesthesia. While most patients exhibit subtoxic fluoride levels, 10% exceed the nephrotoxic threshold (50 µM) [30]. Phenobarbital pretreatment increases defluorination [31]. A major concern is sevoflurane’s instability in soda lime, which generates fluoride and difluoromethyl-trifluorovinyl ether (compound **A**) [32]. Although no clinical adverse effects have been reported, halogenated vinyl compounds can form reactive intermediates with tissue-damaging potential [33].

Conversely, sevoflurane—but not desflurane—has been associated with acute kidney injury (AKI), evidenced by elevated serum creatinine [19,20]. A retrospective study in neurosurgical ICU patients found higher AKI/acute kidney disease (AKD) incidence and reduced survival with sevoflurane versus propofol [34]. Nephrotoxicity was exacerbated by hypotension, diabetes, and coronary artery disease [35,36,37]. Additionally, sevoflurane was associated with poorer renal allograft function compared to isoflurane [38].

## 2. Hypothetical Mechanisms

Sevoflurane induces multi-segmental nephron injury even in healthy volunteers: glomerular (albuminuria), proximal tubular (α-GST elevation, glucosuria), and distal tubular (α-GST elevation) [9]. Six of ten subjects had fluoride levels >100 µmol/L, yet none developed NDI [9]. Renal injury was attributed to compound A exposure (~50 ppm) [19,20].

Premuzic et al. proposed two mechanisms: (1) direct neurogenic modulation by sevoflurane, and (2) intraoperative hypotension as an additive risk factor [34]. Hypertensive patients may be particularly susceptible [36]. Yildirim et al. implicated compound **A** in dose-dependent albuminuria, glucosuria, and enzymuria [37,38,39].

Ray et al. described transient proximal tubulopathy post-sevoflurane, manifesting as glycosuria, phosphaturia, and kaliuresis [40]. We suggest that transient proximal tubule impairment may play a role in the proteinuria and glycosuria described following volatile anesthetic exposure.

### 2.1. Renal Sympathetic Nerve Activity and Volatile Anesthesia

Oliguria during volatile anesthesia remains incompletely understood [41]. While volatile agents generally suppress sympathetic output, isoflurane selectively increases renal sympathetic nerve activity (RSNA) [42]. RSNA activation reduces sodium excretion via

− Afferent arteriolar constriction (↓GFR);
− Renin–angiotensin–aldosterone activation;
− Direct tubular sodium reabsorption [43].

Chronic instrumentation studies in sheep demonstrated that sevoflurane increases RSNA (burst frequency + 105%), causing oliguria (−52%) and natriuresis (−85%) [44]. Taavo et al. confirmed that RSNA—not vasopressin or hypotension—mediates oliguria [45]. Alpha/beta blockade could mitigate RSNA but risks hypotension. While volatile preconditioning benefits cardiopulmonary surgery, no renal protective effects have been observed [46,47,48,49]. Isoflurane reduces renal perfusion more than propofol due to RSNA activation [49].

### 2.2. New Volatile Agents in Transplantation

Acute hepatic failure following general anesthesia with isoflurane represents a rare occurrence in patients without pre-existing liver disease, yet carries significant mortality when it occurs. While an idiosyncratic drug reaction served as our primary diagnostic consideration, the clinical presentation proved particularly perplexing due to both the rapid onset and severity of hepatic failure in the absence of identifiable precipitating factors. Although the differential diagnosis for such presentations remains extensive, we shall focus our discussion on the most clinically relevant etiologies. Contemporary halogenated volatile agents, including isoflurane and sevoflurane, have not demonstrated the same propensity for hepatic dysfunction as halothane [50]. This reduced hepatotoxic potential is attributed to distinct cytochrome-mediated biotransformation pathways employed by newer agents. Specifically, while the CYP2E1 pathway metabolizes approximately 25% of absorbed halothane, it processes only 0.2% of isoflurane and a mere 0.02% of desflurane. Nevertheless, given structural similarities among cytochrome substrates, a theoretical risk persists that oxidative metabolism of any volatile anesthetic could generate reactive complexes capable of eliciting hepatotoxic responses analogous to those observed with halothane [51,52].

Notably, volatile anesthetic-associated hepatic failure has been documented most frequently in patients at age extremes, typically manifesting >48 h post-exposure and often accompanied by additional signs of immunologic or allergic reactions [51,52]. Of particular clinical relevance is the recognized cross-sensitivity between different halogenated anesthetic agents. In the reported case, the patient ultimately required liver transplantation, during which sedation was maintained using propofol [52].

Nieuwenhuijs-Moeke et al. conducted a comparative study evaluating propofol-based versus sevoflurane anesthesia in living donor kidney transplantation recipients. Their findings demonstrated significantly elevated urinary AKI biomarkers on postoperative day two in the sevoflurane group, indicative of renal stress, though no statistically significant difference in graft outcomes was observed between the two anesthetic approaches [53].

### 2.3. Volatile Anesthetics in Non-Cardiac Surgery

Multiple investigations have compared postoperative AKI incidence between propofol and volatile anesthetic regimens. Consistent with our observations, retrospective analyses of nephrectomy, colorectal surgery, and major abdominal procedures have demonstrated reduced AKI incidence with propofol administration [54,55,56,57]. Furthermore, propofol-treated patients exhibited lower concentrations of kidney-specific biomarkers and pro-inflammatory cytokines, along with decreased AKI rates, when compared to sevoflurane anesthesia in both major abdominal and cardiac surgical settings [58,59].

### 2.4. Volatile Anesthetics in Cardiac Surgery

A recent randomized trial evaluated 112 patients undergoing valvular heart surgery receiving either propofol or sevoflurane anesthesia (both supplemented with sufentanil) [58]. The sevoflurane group demonstrated significantly more patients with >25% increases in cystatin C from baseline (39.3% vs. 19.6%, *p* = 0.023) [58]. Postoperatively, AKI developed in 37.5% of sevoflurane-treated patients compared to only 10.7% in the propofol group (*p* = 0.001) [58,59].

### 2.5. Comparative Nephrotoxicity: Desflurane vs. Sevoflurane vs. Isoflurane

An animal study employing a rhabdomyolysis-induced acute tubular necrosis (ATN) model provides insight into the relative nephrotoxic potential of contemporary volatile agents [60,61,62]. Twenty-four rats were randomized into three groups receiving four-hour exposures of either sevoflurane (Sev G), desflurane (Des G), or isoflurane (Iso G), followed by intramuscular glycerol (9 mg/kg) to induce ATN [63]. Biochemical and histopathological assessments revealed that isoflurane caused less severe renal injury compared to both desflurane and sevoflurane in this ATN model [60].

### 2.6. Prevention of Volatile Anesthetic-Induced Acute Kidney Injury

Recent advances in nanotechnology have enabled production of diverse nanoparticle formulations with varying sizes and morphologies [61]. Among these, cerium oxide nanoparticles (CNPs)—derived from the most reactive lanthanide series element—have demonstrated considerable therapeutic potential. While industrial applications include polishing, toxic gas conversion, and sensor/catalyst technologies, medical research has revealed CNPs possess radioprotective, anti-inflammatory, neuroprotective, and anti-ischemic stroke properties. The discovery of intrinsic antioxidant capabilities on nanoparticle surfaces has particularly spurred interest in CNPs for managing oxidative stress-related pathologies [61].

Substantial evidence indicates that inorganic fluoride—generated via hepatic cytochrome-mediated biotransformation of sevoflurane—can exert deleterious effects on both hepatic and renal tissues across species. While numerous studies have characterized sevoflurane’s nephrotoxic potential in humans and animal models, limited data exist regarding protective strategies against anesthesia-induced renal damage. Recent investigations have yielded two key findings: sevoflurane administration (3 h) induced significant tubular epithelial necrosis in rats, accompanied by increased lipid peroxidation and diminished antioxidant capacity, and pretreatment with 0.5 mg/kg CNPs (intraperitoneal) attenuated oxidative damage in renal tubular structures [61].

The observed elevation in malondialdehyde (MDA) levels coupled with reduced antioxidant activity suggests reactive oxygen species play a pivotal role in sevoflurane-mediated tubular injury. CNP pretreatment not only improved renal histoarchitecture (particularly in tubular regions) but also restored oxidant/antioxidant balance [61].

### 2.7. Volatile Anesthesia and Renal Autoregulation

The concept of renal autoregulation warrants careful interpretation, as it does not preclude clinically significant perfusion alterations—rather, such changes seldom result from perfusion pressure variations unless profound hypotension occurs. In reality, local and systemic vasoactive factors predominantly regulate renal perfusion and function [62]. The precise impact of volatile anesthesia on autoregulatory mechanisms remains incompletely understood [63]. Contemporary studies utilizing direct measurement techniques demonstrate that therapeutic doses of inhaled anesthetics reduce renal vascular resistance, thereby maintaining blood flow despite perfusion pressure reductions during anesthesia [62,63]. These hemodynamic alterations typically resolve promptly postoperatively. Notably, even prolonged isoflurane-induced hypotension (mean arterial pressure 60 mmHg) failed to produce measurable postoperative renal dysfunction in one investigation [16].

## 3. Clinical Reality: European Medicines Agency Data

The European Medicines Agency’s EudraVigilance database (current to 15 January 2022) provides critical insights into volatile anesthetic safety profiles. Sevoflurane was associated with 4553 adverse event reports, including 219 renal/urinary disorders (30 cases of NDI). Of note, sevoflurane may have been employed off-label in specific clinical scenarios [64]. By contrast, isoflurane accounted for 1285 adverse events, with only 42 renal/urinary complications (2 NDI cases). These data suggest that routine sevoflurane use—particularly beyond approved indications—may be inadvisable outside rigorously controlled clinical trials [65]. Emerging evidence also implicates elevated fluoride concentrations in chronic kidney disease pathogenesis [66]. We have calculated the proportion of renal/urinary disorders per 1000 adverse events) to strengthen the quantitative analysis of clinical risk differences for sevoflurane and isoflurane. So, the calculated proportion of renal/urinary disorders is respectively 0.2% for sevoflurane and 0.3% for isoflurane.

## 4. Reversibility of Nephrotoxic Effects

While NDI (associated with fluoride levels >50 μmol/L) typically proves reversible, other fluoride-induced renal lesions may cause permanent damage [66]. Similarly, compound A exposure leads to multi-segmental nephron injury—manifesting as glomerular damage (albuminuria), proximal tubular dysfunction (elevated α-GST, glucosuria), and distal tubular involvement (increased α-GST)—which may be irreversible [9]. Regarding more recent literature, the side effects of halothane, desflurane, and sevoflurane have been declared to be minor [67].

## 5. Conclusions

Despite increasing ICU utilization of newer volatile agents like sevoflurane, EudraVigilance data (January 2022) reveal concerning renal safety signals. Among 4553 sevoflurane-associated adverse events, 219 involved renal/urinary disorders (including 30 NDI cases) [64]. The potential for off-label use further complicates risk assessment. In contrast, isoflurane demonstrated markedly fewer renal complications (42/1285 reports, including 2 NDI cases) [65]. Given these findings—and absent robust evidence establishing sevoflurane’s renal safety under well-defined conditions—isoflurane may represent a more judicious choice, particularly for patients with elevated renal risk. In the future, it would be beneficial to utilize cutting-edge target discovery techniques, such as proteomics and PROTAC Probe to further explore and validate the nephrotoxic mechanisms of action of volatile anesthetics [68,69].

## Data Availability

The data that support the findings of this study are available from the corresponding author upon reasonable request.

## References

[1] Pringle H., Mannsell R.C.B., Pringle S. (1905). Clinieal effeets of ether anaesthesia on renal aetivity. Br. Med. J..

[2] Fukazawa K., Lee H.T. (2014). Volatile anesthetics and AKI: Risks, mechanisms, and a potential therapeutic window. J. Am. Soc. Nephrol..

[3] Bovill J.G. (2008). Inhalation anaesthesia: From diethyl ether to xenon. Handbook of Experimental Pharmacology.

[4] Ong Sio L.C.L., Dela Cruz R.G.C., Bautista A.F. (2017). Sevoflurane and renal function: A meta-analysis of randomized trials. Med. Gas. Res..

[5] Haynes R.C., Gilman A.F., RaH T.W., Nies A.S., Taylor P. (1990). Agents affecting calcification. Goodman and Gilman’s. The pharmacological Basis of Therapeutics.

[6] Roman R.J., Carter J.R., North W.C., Kauker M.L. (1977). Renal tubular site of action of fluoride in Fischer 344 rats. Anesthesiology.

[7] Van dyke R., Chenowith M., Poznak A.Y. (1964). Metabolism of volatile anesthetics: I. Conversion in vivo of several anesthetics to ^14^CO_2_ and chloride. Biochem. Pharmacol..

[8] Subcommittee on the National Halothane Study of the Committee on Anesthesia, National Academy of Sciences-National Research Council (1966). Summary of the national halothane study: Possible association between halothane anesthesia and postoperative necrosis. JAMA.

[9] Mazze R.I., Trudell J.R., Cousins M.J. (1971). Methoxyflurane metabolism and renal dysfunction: C1inical correlation in man. Anesthesiology.

[10] Nebert D.W., Adesnik M., Coon M.J. (1987). The P450 gene super-family: Recommended nomenclature. DNA.

[11] Conney A.H. (1967). Pharmacological implications of microsomal enzyme induction. Pharmacol. Rev..

[12] Waxman D.J. (1988). Interactions of hepatic cytochromes P-450 with steroid hormones: Regioselectively and stereospecificity of steroid metabolism and hormonal regulation of rat P-450 enzyme expression. Biochem. Pharmacol..

[13] Pantuck E.J., Pantuck C.B., Conney A.H. (1987). Effect of streptozotocin-induced diabetes in the rat on the metaboJism of fluorinated volatile anesthetics. Anesthesiology.

[14] Jarnberg P.O., De Broe M.E., Porter G.A., Bennett W.M., Verpooten G.A. (1998). Renal toxicity of anesthetic agents. Clinical Nephrotoxins.

[15] Orhan H., Sahin A., Sahin G., Aypar U., Vermeulen N.P. (2013). Urinary lipid and protein oxidation products upon halothane, isoflurane, or sevoflurane anesthesia in humans: Potential biomarkers for a subclinical nephrotoxicity. Biomarkers.

[16] Honore P.M., De Bels D., Barreto Gutierrez L., Redant S., Gallerani A., Boer W. (2019). Sevoflurane and nephrogenic diabetes insipidus on the rise: Copeptin to the rescue?. Crit. Care.

[17] L’Heudé M., Poignant S., Elaroussi D., Espitalier F., Ferrandière M., Laffon M. (2019). Nephrogenic diabetes insipidus associated with prolonged sedation with sevoflurane in the intensive care unit. Br. J. Anaesth..

[18] Cousins M., Mazze R. (1973). Methoxyflurane nephrotoxicity. JAMA.

[19] Eger E.I., Koblin D.D., Bowland T., Ionescu P., Laster M.J., Fang Z., Gong D., Sonner J., Weiskopf R.B. (1997). Nephrotoxicity of sevoflurane versus desflurane anesthesia in volunteers. Anesth. Analg..

[20] Keller K.A., Callan C., Prokocimer P., Delgado-Herrera L., Friedman M.B., Hoffman G.M., Wooding W.L., Cusick P.K., Krasula R.W. (1995). Inhalation toxicity study of a haloalkene degradant of sevoflurane, compound A (PIFE), in Sprague-Dawley rats. Anesthesiology.

[21] Rush G.F., Willis L.R. (1982). Renal tubular effects of sodium fluoride. J. Pharmacol. Exp. Ther..

[22] Chen P.S., Smith F.A., Gardner O.E., O’Brien J.A., Hodge H.C. (1956). Renal clearance of fluoride. Proc. Soc. Exp. Biol..

[23] Carlson C.H., Armstrong W.O., Singer L. (1960). Distribution and excretion of radiofluoride in the human. Proc. Soc. Exp. Biol..

[24] Hosking D.J., Chamberlain M.J. (1972). Studies in man with 18F. Clin. Sci..

[25] Ekstrand J., Ehrnebo M., Boreus L.O. (1978). Fluoride bioavailability after intravenous and oral administration: Importance of renal clearance and urine flow. Clin. Pharmacol. Ther..

[26] Whitford G.M., Pashely D.H., Stringer G.I. (1976). Fluoride renal clearance: A pH-dependent event. Am. J. Physiol..

[27] Ekstrand J., Ehrnebo M., Whitford G.M., Jarnberg P.-O. (1980). Fluoride pharmacokinetics during acid-base changes in man. Eur. J. Clin. Pharmacol..

[28] Jarnberg P.-O., Ekstrand J., Irestedt L. (1981). Renal fluoride excretion and plasma fluoride levels during and after enflurane anesthesia are dependent on urinary pH. Anesthesiology.

[29] Barzel U.S., Jowsey J. (1969). The effect of chronic acid and alkali administration on bone turnover in adult rats. Clin. Sci..

[30] Frink E.J., Ghantous H., Malan T.P. (1992). Plasma inorganic fluoride with sevoflurane anesthesia: Correlation with indices of hepatic and renal function. Anesth. Analg..

[31] Cook T.L., Beppu W.J., Hitt B.A. (1975). A comparison of renal effects and metaboJism of sevoflurane and methoxyflurane in enzyme induced rats. Anesth. Ana1g..

[32] Hanaki C., Fujii K., Morio M., Tashima T. (1987). Decomposition of sevoflurane by sodalime. Hiroshima J. Med. Sci..

[33] Macdonald T.L. (1983). Chernical mechanisms of halocarbon metabolism. CRC Crit. Rev. Toxicol..

[34] Premuzic V., Stambolija V., Lozic M., Kovacevic J., Prelevic V., Peklic M., Scap M., Sekulic A., Basic-Jukic N., Mihaljevic S. (2024). The effect of different anesthetics on the incidence of AKI and AKD after neurosurgical procedures. PLoS ONE.

[35] Davison E., Affleck A., Daratha K.B. (2022). Intraoperative Hypotension and Acute Kidney Injury in Non-cardiac Surgery at a Large Tertiary Care Medical Center. AANA J..

[36] Hallqvist L., Granath F., Huldt E., Bell M. (2018). Intraoperative hypotension is associated with acute kidney injury in noncardiac surgery. Eur. J. Anaesthesiol..

[37] Yildirim M., Kucuk H.F., Demir T., Yakupoglu S., Yavuz A., Ari E. (2015). Early Allograft Function in Renal Transplant Recipients: Is it Affected by Volatile Anesthetics?. Transplant. Proc..

[38] Frink E.J., Malan T., Atlas M., Dominguez L.M., DiNardo J.A., Brown B.R. (1992). Clinical Comparison of Sevoflurane and Isoflurane in Healthy Patients. Anesth. Analg..

[39] Bedford R.F., Ives H.E. (2000). The renal safety of sevoflurane. Anesth. Analg..

[40] Ray E.C., Abdel-Kader K., Bircher N., Rondon-Berrios H. (2015). Case report: Proximal tubule impairment following volatile anesthetic exposure. Physiol. Rep..

[41] Myles P.S., McIlroy D.R., Bellomo R., Wallace S. (2019). Importance of intraoperative oliguria during major abdominal surgery: Findings of the Restrictive versus Liberal Fluid Therapy in Major Abdominal Surgery trial. Br. J. Anaesth..

[42] Iguchi N., Kosaka J., Booth L.C., Iguchi Y., Evans R.G., Bellomo R., May C.N., Lankadeva Y.R. (2019). Renal perfusion, oxygenation, and sympathetic nerve activity during volatile or intravenous general anaesthesia in sheep. Br. J. Anaesth..

[43] Osborn J., Tyshynsky R., Vulchanova L. (2021). Function of renal nerves in kidney physiology and pathophysiology. Annu. Rev. Physiol..

[44] Hart E.C., Head G.A., Carter J.R., Wallin B.G., May C.N., Hamza S.M., Hall J.E., Charkoudian N., Osborn J.W. (2017). Recording sympathetic nerve activity in conscious humans and other mammals: Guidelines and the road to standardization. Am. J. Physiol. Heart Circulat Physiol..

[45] Taavo M., Rundgren M., Frykholm P., Larsson A., Franzén S., Vargmar K., Valarcher J.F., DiBona G.F., Frithiof R. (2021). Role of renal sympathetic nerve activity in volatile aneshesia’s effect on renal excretory function. Function.

[46] Frithiof R., Soehnlein O., Eriksson S., Fenhammar J., Hjelmqvist H., Lindbom L., Rundgren M. (2011). The effects of isoflurane anesthesia andmechanical ventilation on renal function during endotoxemia. Acta Anaesthesiol. Scand..

[47] Garcia C., Julier K., Bestmann L., Zollinger A., von Segesser L.K., Pasch T., Spahn D.R., Zaugg M. (2005). Preconditioning with sevofluranedecreases PECAM-1 expression and improves one-year cardiovas-cular outcome in coronary artery bypass graft surgery. Br. J. Anaesth..

[48] Sindhvananda W., Phisaiphun K., Prapongsena P. (2013). No renal protection from volatile-anesthetic preconditioning in open heart surgery. J. Anesth..

[49] Dharmalingam S.K., Amirtharaj G.J., Ramachandran A., Korula M. (2021). Volatile anesthetic preconditioning modulates oxidative stress and ni-tric oxide in patients undergoing coronary artery bypass grafting. Ann. Card. Anaesth..

[50] Meldrum D.J., Griffiths R., Kenna J.G. (1998). Gallstones and isoflurane hepatitis. Anaesthesia.

[51] Ihtiyar E., Algin C., Haciolu A., Isiksoy S. (2006). Fatal isoflurane hepatotoxicity without re-exposure. Indian J. Gastroenterol..

[52] Peiris L.J., Agrawal A., Morris J.E., Basnyat P.S. (2012). Isoflurane hepatitis-induced liver failure: A case report. J. Clin. Anesth..

[53] Nieuwenhuijs-Moeke G.J., Nieuwenhuijs V.B., Seelen M.A.J., Berger S.P., van den Heuvel M.C., Burgerhof J.G.M., Ottens P.J., Ploeg R.J., Leuvenink H.G.D., Struys M.M.R.F. (2017). Propofol-based anaesthesia versus sevoflurane-based anaesthesia for living donor kidney transplantation: Results of the VAPOR-1 randomized controlled trial. Br. J. Anaesth..

[54] Lee H.-J., Bae J., Kwon Y., Jang H.S., Yoo S., Jeong C.W., Kim J.-T., Kim W.H. (2019). General anesthetic agents and renal function after nephrectomy. J. Clin. Med..

[55] Bang J.Y., Lee J., Oh J., Song J.G., Hwang G.S. (2016). The influence of propofol and sevoflurane on acute kidney injury after colorectal surgery: A retrospective cohort study. Anesth. Analg..

[56] Kim B.R., Yoon S., Song G.Y., Lee S., Bahk J.H., Nam K. (2021). The impact of total intravenous anesthesia versus inhalation anesthesia on acute kidney injury after major abdominal surgery: A propensity score analysis. J. Anesth..

[57] Kwon J.-H., Park J., Lee S.-H., Oh A.-R., Lee J.-H., Min J.J. (2019). Effects of volatile versus total intravenous anesthesia on occurrence of myocardial injury after non-cardiac surgery. J. Clin. Med..

[58] Ammar A.S., Mahmoud K.M. (2016). Comparative effect of propofol versus sevoflurane on renal ischemia/reperfusion injury after elective open abdominal aortic aneurysm repair. Saudi J. Anaesth..

[59] Yoo Y.C., Shim J.K., Song Y., Yang S.Y., Kwak Y.L. (2014). Anesthetics influence the incidence of acute kidney injury following valvular heart surgery. Kidney Int..

[60] Erdem A.F., Ligaz A., Yuksek M.S., Gürsan N., Atalay C. (2007). The Effects of Isoflurane, Sevoflurane and Desflurane Anesthesia on the Glyserol Model of Rhabdomyolysis-Induced Acute Renal Failure in Rats. Eurasian J. Med..

[61] Sivgin V., Kasikara H., Kucuk A., Inan H.M., Gok G., Arslan M., Kıran M.M., Ozturk L. (2023). The Effects of Cerium Oxide on Sevoflurane Anesthesia and its Relationship to Renal Injury in Rats. Gaz. Med. J..

[62] Hollenberg N.K., Zelis R. (1975). The renal circulation. The Peripheral Circulations.

[63] Groves N.D., Leach K.G., Rosen M. (1990). Effects of halothane, enflurane and isoflurane anaesthesia on renal plasma flow. Br. J. Anaesth..

[64] Sneyd J.R. (2022). Avoiding kidney damage in ICU sedation with sevoflurane: Use isoflurane instead. Br. J. Anaesth..

[65] Honore P.M., Bousbiat I., Perriens E., Blackman S. (2023). Letter to the Editor: “Prolonged sedation with sevoflurane in comparison to intravenous sedation in critically ill patients—A randomized controlled trial”. J. Crit. Care.

[66] Wimalawansa S.J. (2016). The role of ions, heavy metals, fluoride, and agrochemicals: Critical evaluation of potential aetiological factors of chronic kidney disease of multifactorial origin (CKDmfo/CKDu) and recommendations for its eradication. Environ. Geochem. Health.

[67] Yaxley J. (2025). Anaesthesia in chronic dialysis patients: A narrative review. World J. Crit. Care Med..

[68] Yan S., Zhang G., Luo W., Xu M., Peng R., Du Z., Liu Y., Bai Z., Xiao X., Qin S. (2024). PROTAC technology: From drug development to probe technology for target deconvolution. Eur. J. Med. Chem..

[69] Qin S., Xiao X. (2025). Key advances and application prospects of PROTAC technologies in the next 5 years. Future Med. Chem..

